# COVID-19: Short and Long-Term Effects of Hospitalization on Muscular Weakness in the Elderly

**DOI:** 10.3390/ijerph17238715

**Published:** 2020-11-24

**Authors:** Lucía Sagarra-Romero, Andrea Viñas-Barros

**Affiliations:** 1 Faculty of Health Sciences, Universidad San Jorge, 50830 Zaragoza, Spain; 2 Hospital General San Jorge, 22004 Huesca, Spain; avinnas@salud.aragon.es

**Keywords:** COVID-19, older adult, bed rest, muscle weakness, functional decline

## Abstract

The COVID-19 pandemic has recently been the cause of a global public health emergency. Frequently, elderly patients experience a marked loss of muscle mass and strength during hospitalization, resulting in a significant functional decline. This paper describes the impact of prolonged immobilization and current pharmacological treatments on muscular metabolism. In addition, the scientific evidence for an early strength intervention, neuromuscular electrical stimulation or the application of heat therapy during hospitalization to help prevent COVID-19 functional sequels is analyzed. This review remarks the need to: (1) determine which potential pharmacological interventions have a negative impact on muscle quality and quantity; (2) define a feasible and reliable pharmacological protocol to achieve a balance between desired and undesired medication effects in the treatment of this novel disease; (3) implement practical strategies to reduce muscle weakness during bed rest hospitalization and (4) develop a specific, early and safe protocol-based care of functional interventions for older adults affected by COVID-19 during and after hospitalization.

## 1. Introduction

The World Health Organization (WHO) has declared the novel coronavirus SARS-CoV-2 (hereinafter COVID-19) disease a global pandemic. It menaces current lifestyle and is prompting irreversible consequences in health, economic and social stability. This novel coronavirus can produce severe disease symptoms among people of all ages; however, it has been evidenced that older adults with multi-morbidity are at the highest risk of poor prognosis due to COVID-19 [1]. In fact, old age has been reported as a significant independent predictor of mortality in the COVID-19 pandemic. For example, the mortality rate for COVID-19 infection on the Chinese population in the >80 years age group was 21.9% compared with 1.3% in the 50–59 age group [2,3].

Common complications from COVID-19 infection are acute viral pneumonitis evolving to acute respiratory distress syndrome (ARDS), acute kidney injury (AKI), proinflammatory hypercoagulable state with thromboembolic events, sepsis and cardiac injury. Complications are believed to derive from a pro-inflammatory state with cytokine release [4,5]. 

It is noteworthy that COVID-19 infected patients tend to experience a prolonged hospitalization or intensive care unit (ICU) stay, with an average of three weeks in the ICU [6]. Commonly, bed rest is prescribed in infected patients in order to minimize the metabolic demand and orientate resources towards the recovery process. However, it has been evidenced that long periods of immobilization and rest in hospital and ICU produce a negative impact on several body systems. As an example, a period of four to six weeks of bed rest has been shown to cause muscle wasting, loss of muscle force generation capacity (6% to 40% muscle strength) and changes in contractile proteins (muscle protein turnover) among others [7]. 

**Figure 1:**
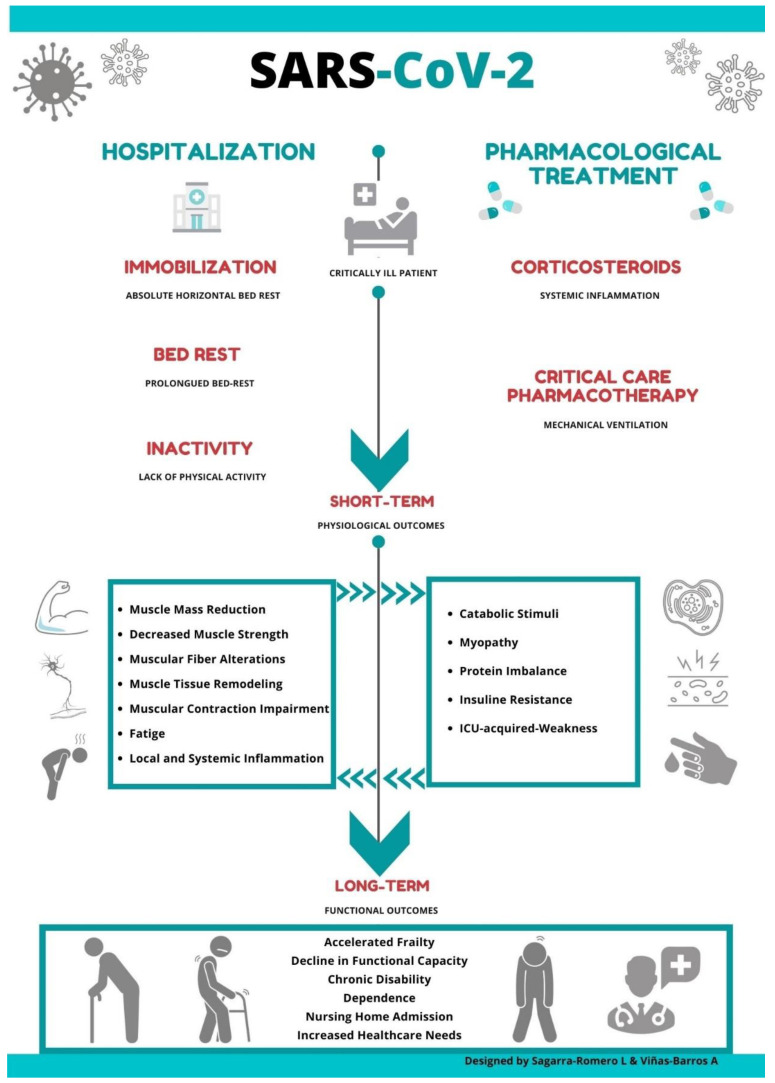
The effect of drug therapy treatment and long periods of bed rest on muscular weakness in elderly patients infected with COVID-19.

The sequelae of prolonged immobility on SARS patients’ musculoskeletal system has been previously documented [8]; however, little is known about the consequences in muscular function and deterioration associated with this novel coronavirus. Thus, it is possible to suggest that the convergence of certain drug therapy treatment and long periods of bed rest might negatively impact muscular weakness, accelerating a functional decline in elderly patients infected with COVID-19 (Figure 1).

It seems important to keep in mind that the aging process is accompanied by an inherent sarcopenic process, characterized by an accelerated loss of muscle mass. Hence, the risk of suffering sarcopenia and fragility increases with age [9]. Unfortunately, there is no evidence to date about the impact of infection for COVID-19 on this degenerative process and it is unclear whether patients under the age of 70 years will experience premature frailty as a consequence of the disease. In this novel coronavirus scenario, Abbatecola et al. [10] have recently coined a new concept named ‘COVID spiraling frailty syndrome’ as a crucial entity with a negative impact on elderly people, derived from the convergence of factors such as advanced age and the presence of comorbidities. Furthermore, correlations between the average hospital length of stay, doses of the pharmacological treatments and the consequent neuromuscular damage need to be explored further.

Thus, the purpose of this paper was to (1) determine which potential pharmacological interventions have a negative impact on muscle quality and quantity; (2) define a feasible and reliable pharmacological protocol to achieve a balance between desired and undesired medication effects in the treatment of this novel disease; (3) implement practical strategies to reduce muscle weakness during bed rest hospitalization and (4) develop a specific, early and safe protocol-based care of functional interventions for older adults affected by COVID-19 during and after hospitalization.

## 2. COVID-19: Impact of Pharmacological Treatment on Muscle Metabolism

Empirical therapies including glucocorticosteroids as immune-modulatory agents are being prescribed [11]. Moreover, in COVID-19 ICU-admitted patients, ventilatory, renal support and hemodynamic stability are pursued under a regime that includes high-flow nasal cannula, invasive mechanical ventilation, vasoactive and continuous renal replacement therapy (CRRT). Noradrenalin is currently recommended as a first choice followed by vasopressin [12]. Some of these drugs have a known interference with protein synthesis and muscular metabolism and may potentially contribute to physical function impairment in survivors of COVID-19 infection. For example, systemic glucocorticosteroids (GCs) have well-known anti-inflammatory and immunosuppressive properties. Therapy with GCs is applied to clinical practice in order to inhibit a cytokine storm and manage inflammatory-induced lung injury in COVID-19 patients [13]. Different treatment schedules are used depending on the patient’s characteristics, clinical severity and the preference of the physician. In the clinical setting, the standard methylprednisolone-equivalent dosages range from 0.5–1 mg/kg with a variable duration depending on the clinical response. The results from a preliminary trial suggest the benefit of using a low dose of dexamethasone, 6 mg/day, or 32 mg/day of methylprednisolone as an equivalent, in severely ill COVID-19 patients [14]. However, high doses (>1–1.5 mg/kg/day of methylprednisolone) are frequently administered in the clinical setting. For example, pulses of methylprednisolone ranging from 125 to 1000 mg/day during one to five days are commonly used with severe patients. Scientific evidence and clinical experience support this practice too [15].

The delicate clinical condition due to the risk of respiratory failure warrants its use. However, as they usually induce a wide range of side effects, adverse outcomes derived from their use are observed and should be managed accordingly.

Past studies have suggested that the use of systemic corticoids for treating acute lung injury during the 2003 SARS-CoV emergency contributed to muscular weakness and decreased functional capacity observed in survivors at follow-up visits [16]. It is important to note that corticosteroid therapy can also exert muscular damage through an impairment in the electrical excitability of muscle fibers, a decrease in the number of thick filaments and a reduction in anabolic protein synthesis along with an increased protein degradation [17]. Indeed, muscular weakness is a well-known chronic side effect observed in patients undergoing a long-term treatment with GCs. It has also been evidenced that the administration of GCs over a short period of time can induce an early-onset myopathy in critically ill patients, which is characterized by a progressive weakening of several muscle groups.

Steroid-induced myopathy is most frequently observed in patients treated with >10mg/day of prednisone or its equivalent for a few weeks [18]. Generally, the higher the dose, the greater the likelihood of developing histological changes more rapidly. A few authors have reported acute myopathy even with standard doses (40–60 mg/day of prednisone) and independently from the route of administration or the kind of corticosteroid [19]. As an example, Hanson et al. [20] showed findings of acute myopathy after five days of treatment with a dose equivalent of 60 mg per day of methylprednisolone. Furthermore, old age and several ageing mechanisms may increase the risk of steroid-induced myopathy. Thus, it is plausible that treatment with GCs may exert a role in muscle deterioration in older COVID-19 survivors. Unfortunately, further clinical investigations are needed to better define the relationship between dose-response used in COVID-19 infected patients and the damage exerted to muscular quality.

Finally, it seems important to remark that certain pharmacological interventions used in the ICU environment may further exacerbate muscular damage. For example, the use of B-adrenergic vasoactive agents such as norepinephrine to treat circulatory failure has been independently associated with ICU-acquired weakness [21]. The use of neuromuscular blocking agents during mechanical ventilation has been classically considered as a risk factor; however, it is not clear to date if they actually play a role in the development of muscular weakness [22].

## 3. COVID-19: Impact of Hospitalization and ICU on the Musculoskeletal System

It has been evidenced that the hospitalization rates for COVID-19 increase with age and that older adults are at the highest risk of hospital admission [23]. For many years, the negative functional consequences of prolonged hospitalization have been well recognized. In the context of COVID-19, recent epidemiological studies have reported an average in-hospital length of stay of 20 days [24,25] with an average ICU stay of three weeks [6]. This seems important because the number of days of bed rest during hospitalization or ICU stay is today regarded as a predictive factor for the deterioration of neuromuscular properties [26]. It has been evidenced that in young people, a bedridden period of three weeks has a greater negative impact on functional capability than 40 years of aging [27]. Remarkably, even a short period of absolute rest (up to 10 days) may trigger skeletal muscle wasting. Kortebein et al. [28] found a substantial loss of muscle strength and power (knee extension *p* = 0.004, knee flexion *p* = 0.003 and stair ascent power *p* = 0.01) after 10 days of bed rest in healthy elderly people (60–85 years old).

It should be noted that older patients are especially vulnerable to these changes, with a higher risk of functional dependence loss and cognitive decline after discharge [29]. In fact, it is likely that pre-frail patients admitted to ICU due to critical COVID-19 complications who undergo prolonged body immobilization (more than 10 days) experience a bilateral muscle weakness and decreased muscle strength with devastating consequences on functional capacity [26]. 

Ageing is accompanied by an inherent sarcopenic process characterized by an accelerated loss of muscle mass and function; however, prolonged bed rest with muscular disuse can precipitate this “catabolic crisis” causing skeletal muscle atrophy (low muscle mass and low muscle function) [30]. Thus, a premature functional disability is expected.

Studies have shown how absolute rest can lead to various adverse effects such as changes in total muscle mass, metabolic activity, muscle denervation and a loss of contractile force with increasing fatigue and reduced muscle strength [31,32] (Figure 1).

The aetiology of the coronavirus-related muscle weakness in hospitalized older patients seems to involve several interdependent processes. Firstly, a long period of bed rest seems to be predominant in COVID-19 patients. It is known that becoming temporarily bedridden with a lack of muscular weight-bearing activities is usually followed by an alteration in muscular protein homeostasis. This imbalance may occur quickly and be secondary to an accelerated muscle protein breakdown and a suppression of muscle protein synthesis [33]. This catabolic process is mediated by neurohormonal disorders and systemic inflammation. As a result, alterations in muscle mass and structure are commonly observed among these patients. Moreover, a reduction in the strength of fast-twitch fibers compared with slow-twitch fibers is also likely to happen with a consequent deterioration in resistance capacity [26]. Secondly, other factors such as severe illness, sepsis, mechanical ventilation, parenteral nutrition and certain drug therapies involved in COVID-19 treatment might further accelerate this weakening process [34]. Additionally, other mechanisms have a compounding effect on neuromuscular performance impairment. For example, becoming temporarily bedridden has been further associated with muscle fiber denervation, neuromuscular junction damage and membrane hypoexcitability [26].

Other factors such as cellular bioenergetics are also involved. It is believed that mitochondrial dysfunction may play an important role in the physical function impairment observed in COVID-19 patients. Muscle mitochondria deterioration and therefore the muscle’s capacity to utilize O_2_ is only one part (and not the most important one) of the story. The main cause of the acute reduction of VO_2_max during bed rest is a marked reduction of cardiac output, i.e., of the capacity to deliver O_2_ to the working muscles. Cardiac output is markedly reduced because of a reduction in pre-load (to which the loss of muscle mass/muscle pump contributes substantially) and contractility of the heart. While muscle deterioration takes weeks to happen, at least in healthy individuals, the loss of VO_2_max resulting from a drop in cardiac output is very rapid (1% loss per day from day one of bed rest) [35].

Absolute bed rest, mechanical ventilation and the hyperinflammation status in elder patients can trigger a reduction in muscular mitochondrial content and a decrease in phosphorylation enzyme activity. Patients’ fatigue resistance and endurance capacity reduction after recovery is attributed to these processes [36,37,38].

Remarkably, clinical practice and research articles point out that the virus itself can cause myopathic changes unrelated to pharmacological treatment or a critical illness state. Leung et al. reported muscle atrophy and focal necrosis in infected patients based on muscular tissue samples, suggesting that a few of these changes may be the consequence of the activation of local cytokine-mediated pathways alone [39]. There is increasing evidence that COVID-19 patients typically present with general weakness and myalgias that may persist even for weeks after the acute phase. Surprisingly, in many ambulatory and hospitalized patients, acute skeletal muscle damage is usually presented as the initial clinical manifestation in some COVID-19 patients, with high concentrations of creatine kinase [40]. Furthermore, a clinical study during the SARS epidemic found elevated concentrations of serum creatine kinase in 32% of subjects [41].

The evaluation of muscle weakness is recommended to tailor the intervention strategies and to assess their effect. Muscle weakness covers both muscle function and muscle structure. Muscle function is underlined by the three concepts of muscle strength, muscle power and muscle endurance. Muscle strength is measured routinely in clinical settings especially for the diagnosis of sarcopenia and frailty. Handgrip strength is the most used choice for the assessment of overall muscle strength [42]. It is an easy measure and only requires a handheld dynamometer. It is recognized to be easily applicable both in research and in clinical settings [43]. Additionally, muscle structure, muscle mass and muscle quality can be quickly assessed at the bedside with an ultrasound technique [44,45].

This scenario highlights the necessity of awareness of the harmful effects of prolonged rest and the factors aforementioned in order to prevent the functional decline in frail and non-frail ICU-admitted or hospitalized patients who are infected by COVID-19.

## 4. COVID-19: Non-Pharmacological Strategies

Two phases have been identified in hospitalized patients. The first acute phase is characterized by a respiratory syndrome and the second, more prolonged phase, prevailing the neuromotor sequelae and impaired functional status. Muscular structure and neuromuscular function decrease exponentially with a combination of pharmacological treatment, extended bed rest and mechanical ventilation, as previously mentioned. 

Preventative non-pharmacological actions have been proven to positively impact long-stay patients; however, early intervention is frequently underutilized. The negative consequences in health of this scenario highlight the importance of an early health intervention in elderly people hospitalized with COVID-19.

Before implementing any interventions requiring close patient contact, a protocol must be established and adhered to in order to prevent virus transmission. For instance, all masks and materials, namely, machines, elastic bands and dumbbells, must be sterilized before and after use. Both the patient and the healthcare professional must follow general hygiene measures including using disposable tissues when coughing or sneezing, washing and disinfecting hands thoroughly and maintaining physical distance when possible. It is also imperative that the healthcare professionals use single use disposable gloves, wear a disposable fluid-resistant coverall/gown, a face mask respirator (FFP2/FFP3), eye/face protection, overshoes and protective headgear. In addition, cleaning and disinfection procedures should be applied in hospital rooms regularly in order to prevent and control the spread of pathogens. Specifically, health services should implement environment restructuring measures (separate equipment, create ground marks and deactivate digital access), provide hand sanitizers or washbasins thoroughly inside facilities, schedule specific cleaning times, schedule training times, encourage the use of individual bottles and observe air conditioning specification with respect to air exchange [46,47].

The following section provides scientific evidence of early functional beneficial interventions among hospitalized older adults. 

### 4.1. Early Strength Intervention

Physical function reduction in patients hospitalized with chronic diseases is preventable; advanced age, acute and chronic disease and illness, functional limitations and deconditioning all contribute to older adults’ vulnerability to functional decline during hospitalization [48]. As a result, the benefits of strength interventions during the hospital stay have been previously evidenced [49]. An early intervention during the hospitalization period has been associated with a better recovery [50]. Kalish et at. [51] found positive outcomes on physical, psychological and social factors after mobilizing hospitalized adults after the review of 36 studies.

Among the elderly, despite there not being a standardized exercise strength protocol, an intervention of low to moderate resistance training appears to be the most effective to fight against the loss of muscle mass, strength and functional capacity [30]. Courtney et al. [52], in a study carried out with hospitalized elderly patients (>65 years old), found improvements after an exercise intervention. As part of the study, a feasible resistance exercise program was developed aimed at strengthening the muscles of the lower limbs such as gluteus, quadriceps, hip flexors and hip adductors/abductors, among others [53]. It included several resistance band exercises completed with 2–3 sets of 10 repetitions (3–5 seconds contractions/rep).

The positive effects of an early intervention in patients hospitalized with pneumonia have been also been reported [54]. Therefore, an early, controlled muscle mobilization and resistance training is utterly important among older adults hospitalized because of COVID-19 to counteract the negative impact on the neuromotor system and physical function.

Ideally, it should be implemented as soon as possible to obtain the maximum benefit. However, during the COVID-19 acute phase, the risk of rapid desaturation lies with changes in position that can potentially alter the ventilation/perfusion ratio and hemodynamic stability. Consequently, much consideration has been given to when to start these interventions, i.e., medical boards in various countries generally advise against these measures in the acute phase especially in critical patients [55]. Nevertheless, a few institutions tend to start them during mechanical ventilation [56].

A recent scientific statement emphasized the importance of gradually starting rehabilitation therapy for patients affected by COVID-19 to avoid acute hypoxemia [6]. In patients with a severe respiratory condition reflected by bilateral interstitial radiological signs with PaO_2_/FiO_2_ < 300 or a low Glasgow Coma Scale level, a progressive introduction of passive mobilization exercises and frequent position changes against gravity should be attempted to prevent deconditioning. Clinical parameters (temperature, SpO_2_, SpO_2_/FiO_2_, dyspnea, respiratory rate, thoracic-abdominal dynamics, blood pressure, heart rate, peripheral perfusion and Glasgow Coma Scale) should be thoroughly monitored. Criteria for interrupting the therapy include a respiratory rate >40 or <5 breaths/min, SpO_2_ < 88%, systolic blood pressure >200 or <90, diastolic blood pressure >110 or <60, heart rate >110 or <40, instauration of arrhythmias or angor pectoris, peripheral circulation deficits, agitation and neurological impairment [57]. 

Routines including limb mobilization are essential to avoid side effects on the musculoskeletal system (pressure ulcers, critical illness myopathy, plantar flexor contractures or heterotopic ossification) especially for those patients after a prolonged pronation passive mobilization therapy following ARDS [58]. A few institutions employ lower body ergometers to achieve passive leg movements; this could be of special interest wherever the nurse-to-patient ratio may interfere with clinical assistance [56].

When clinical improvement is attained, a progressive resistance training (i.e., low to moderate intensity) performed with resistance bands and free weights of body weight in the post-acute phase can be considered as a safe intervention for the elderly. Active mobilization (simple bed exercises, bedside sitting and standing), accompanied by progressive muscular strengthening should be considered whenever the clinical situation allows it [59].

### 4.2. Neuromuscular Electrical Stimulation

The use of electrical stimulation is an emerging strategy of intervention with positive effects in seriously ill hospitalized patients who are unable to perform resistance exercises. The effectiveness of this therapy has been evidenced in immobilized patients after a spinal cord injury, congestive heart failure and chronic obstructive lung disease [60]. In a recent paper, it has been found that daily neuromuscular electrical muscle activation (seven days a week) induced muscle mass preservation in hospitalized geriatric patients; however, the results showed limited evidence of effects on the whole body functional capacity [61]. Rodriguez et al. [62] found positive effects on the strength of biceps and quadriceps after a 13-day intervention of neuromuscular electrical simulation in septic patients. Contrarily, Zinglersen et al. [63] found positive changes on functional measures in a combined short intervention in hospitalized elderly patients that were not attributable to neuromuscular electrical stimulation.

Despite the apparent safety of electrical stimulations in the initial stages of treatment, it is highly important to develop effective protocols of interventions for hospitalized elders that can be combined with either dynamic mobilization or functional strength exercises.

### 4.3. Heat Therapy

The benefits of thermotherapy interventions have been previously evidenced in animal, in vitro and human studies. The application of heat on the whole body contributes to muscle recovery attenuating cellular damage and protein degradation [64]. Moreover, heat therapy induces capillarization, muscle hypertrophy and mitochondrial biogenesis in skeletal muscle models [65]. Hesketh et al. [66] found positive microvascular adaptations after a six week intervention of heat therapy in young sedentary participants; however, no changes on muscle architecture (mitochondrial density) were found. Heat therapy can be useful for muscular pain and body inflammatory responses producing an analgesic effect for patients during hospitalization and ICU stays [67]. 

The novel scenario of COVID-19 and the constellation of the explained features should alert healthcare professionals to the possibility of an accelerated decline on physical function among the elderly. The use of concomitant interventions might be one of the major challenges for healthcare providers in order to ameliorate the functional decline of elderly patients, enhancing their recovery process and improving global survival rates. 

## 5. COVID-19: Long-Term Consequences

The long-term consequences and sequelae of COVID-19 are still unclear. The interest in long-term outcomes after recovery from SARS-CoV and MERS-CoV global epidemics has increased over the last years. Clinical investigations have reported that in the following months after SARS-CoV discharge, elderly patients experienced a significant functional decline, reduced musculoskeletal fitness, poor cardiorespiratory fitness and a lower quality of life than control subjects (shortly after discharge and at 24 months follow-up) [68,69]. While the literature has not yet fully revealed the effect on people surviving the COVID-19 pandemic, a few authors suggest that elderly survivors will most likely have reduced health derived from both the cardiorespiratory dysfunction sequelae and the ICU or hospitalization stay [70], as seen in previous epidemics. A recent longitudinal study carried out in Wuhan has shown that three months after hospital discharge, 28.3% and 4.5% of survivors suffered from physical decline and myalgias, respectively [71]. 

Interestingly, it has been observed in clinical practice that after overcoming COVID-19 disease, both in ambulatory care and hospital settings, a vast number of elderly patients have reported prolonged general weakness and muscular fatigue for several days and weeks. This is especially noticeable in ICU survivors as they encounter a significant loss of muscle thickness and appreciable motor incoordination [72]. Special attention has been paid to the release of pro-inflammatory cytokines on the potential damage inflicted to the myoneural junction and muscle architecture.

Survivors after prolonged hospitalization and ICU stays generally sustain some degree of functional disability even years after the initial insult [73]. Motor dependence has been evidenced in 60–70% of patients after an ICU stay [74]. In addition, these patients frequently have a decreased ability to walk, impaired balance and reduced mobility as well as a neurocognitive decline, leading to a worsening in instrumental activities (IADL) and activities of daily living (ADL) performance [75].

Usually, a longer period of immobilization comes with a greater functional loss and slower rehabilitation after discharge. Moreover, the length of stay has been shown to have a positive correlation with a risk of falls and injuries [76]. Furthermore, the inflammatory process that frequently accompanies a more severe disease course needing bed rest further exacerbates this process, prompting frailty in pre-frail adults following a more rapid change in muscle wasting, neuromuscular damage and cognitive impairment [29,77].

These long-term consequences are expected to come at a significant additional cost with increased nursing home admission and hospital readmissions. Costs derived from pharmacotherapy, medical complications management and neuropsychological outcomes should be considered [73]. In relation to the above, it is also important to consider the wellbeing of caregivers such as burnout, time off work and financial hardships [78].

## 6. Conclusions

Older people diagnosed with a COVID-19 infection are at a higher risk of severe muscle weakness and atrophy that may have negative consequences on functional disability.

Therefore, it is extremely important to: (1) determine which potential pharmacological interventions have a negative impact on muscle quality and quantity; (2) define a feasible and reliable pharmacological protocol to achieve a balance between desired and undesired medication effects in the treatment of this novel disease; (3) implement practical strategies to reduce muscle weakness during bed rest hospitalization and (4) develop a specific, early and safe protocol-based care of functional interventions for older adults affected by COVID-19 during and after hospitalization.
